# Zr-gallic acid based metal organic compound as adsorbent for extraction of uranium(vi) from nitrate solution: adsorption behaviors and mechanisms

**DOI:** 10.1039/d5ra05880e

**Published:** 2026-01-14

**Authors:** Ahmed M. Masoud

**Affiliations:** a Nuclear Materials Authority P.O. Box 530, El Maddi Cairo Egypt chemmaso010@hotmail.com

## Abstract

In this study, a zirconium-gallic acid metal–organic compound (Zr-GA) was synthesized *via* a hydrothermal method and evaluated as a high-performance adsorbent for uranium(vi) removal from aqueous solutions. Comprehensive physicochemical characterization confirmed the formation of a mesoporous sorbent with a highly functionalized surface rich in oxygen-donor groups such as carboxylates and phenolics, enabling a high density of reactive binding sites. Batch experiments were conducted to assess the effects of pH, dosage, contact time, temperature, and uranium concentration, with all variables showing strong influence on sorption efficiency under 0.1 M nitric acid conditions. Kinetic studies followed the pseudo-second-order model, suggesting a chemisorption-controlled mechanism, while intraparticle and Boyd diffusion analyses revealed a two-stage process dominated by film diffusion followed by internal pore transport. Isotherm modeling confirmed monolayer sorption behavior with a maximum capacity of 107.2 mg g^−1^, as best described by the Langmuir and Sips models. Thermodynamic data (Δ*G*° < 0, Δ*H*° = −71.4 kJ mol^−1^, Δ*S*° < 0) revealed a spontaneous, exothermic, and entropy-reducing process, consistent with strong surface complexation. Desorption studies showed >94% U(vi) recovery using dilute acids, with the Zr-GA retaining >90% efficiency over five reuse cycles. Application to real radioactive liquid waste confirmed the material's selectivity and robustness, achieving 89.7% U(vi) removal under acidic, competitive conditions. These results highlight Zr-GA as a structurally stable, regenerable, and highly effective adsorbent for uranium remediation in both laboratory and field-relevant aqueous systems.

## Abstract 2

Zr–GA efficiently removes U(vi) from aqueous solution *via* inner-sphere surface complexation with oxygen-donor sites, exhibiting high selectivity, monolayer adsorption, and chemisorption behavior.

## Graphical abstract

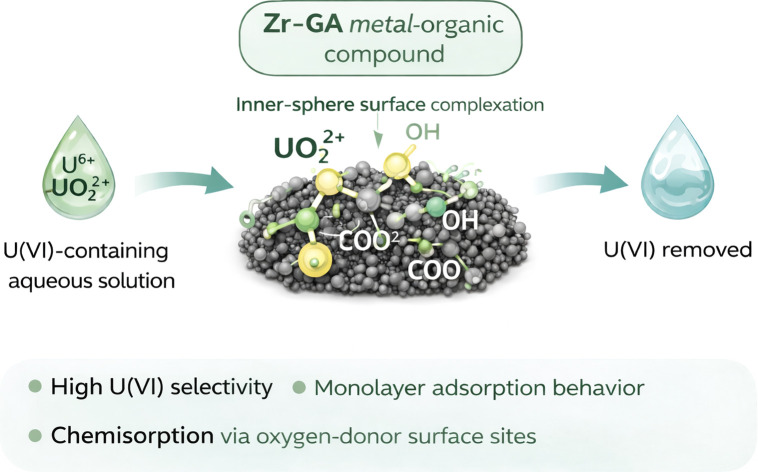

## 1. Introduction

The efficient extraction of uranium from aqueous systems continues to be a critical issue in the nuclear industry and environmental protection sectors. Uranium(vi), which predominates in oxidizing aqueous environments as the uranyl ion (UO_2_^2+^), is not only radiotoxic but also highly soluble and mobile in groundwater and industrial effluents.^1^ This mobility makes it especially problematic in nuclear fuel cycle operations, particularly during fuel reprocessing and the treatment of contaminated wastewater streams.^2,3^ Given the growing global reliance on nuclear energy and the parallel emphasis on environmental sustainability, there is an urgent need to develop advanced materials that can selectively and efficiently capture U(vi) under complex chemical conditions—such as those found in nitrate-rich media resulting from nitric acid leaching or reprocessing operations.^4,5^

Several methods have been explored for the extraction of uranium(vi) from aqueous media, including solvent extraction,^6^ ion exchange,^7^ membrane technologies,^8^ electrochemical approaches,^9^ and precipitation.^10^ However, adsorption has emerged as one of the most promising techniques due to its simplicity, cost-efficiency, and adaptability to various water chemistries.^11–13^ This is especially relevant in nitrate-rich environments, where selective removal of uranyl ions can be challenging.^14^

In recent years, metal–organic materials—including metal–organic frameworks and related coordination solids—have gained considerable attention as versatile platforms for capturing heavy metals and radionuclides, particularly uranium.^15^ Classical MOFs are typically described as crystalline hybrid networks assembled from metal nodes and organic linkers, giving rise to ordered porous architectures with tunable chemical functionality and, in many cases, high surface areas.^16^ Zirconium-based MOFs (*e.g.*, UiO-type and MOF-808 analogues) have been particularly investigated due to their high chemical robustness under aqueous and acidic environments.^15–17^ Nevertheless, it is increasingly recognized that many zirconium-organic coordination materials prepared under practical conditions may exhibit limited long-range order or mixed textural porosity, yet still provide high adsorption performance when they present abundant accessible binding functionalities and chemical stability.^18^

Incorporating functional organic ligands capable of strong coordination with uranium ions is a key strategy to improve adsorption selectivity and affinity. Gallic acid (3,4,5-trihydroxybenzoic acid), a naturally occurring phenolic acid, offers multiple oxygen-donor sites, including three hydroxyl groups and one carboxyl group, which can participate in coordination and surface complexation with metal ions.^19^ These functionalities enable effective interaction with actinide species through ligand exchange/complexation, hydrogen bonding, and electrostatic attraction, depending on solution chemistry.^19–21^ Importantly, gallic acid can form zirconium–oxygen–organic coordination motifs that yield chemically stable zirconium–polyphenol metal–organic compounds, providing high densities of oxygenated binding sites without necessarily requiring a fully crystalline materials architecture.^22,23^ The benign nature, low cost, and sustainability of gallic acid further support its use in developing greener adsorbents for nuclear-waste-related water treatment.

Despite promising advances in metal–organic materials for radionuclide removal, several challenges remain. A critical gap is the limited mechanistic understanding of U(vi) interactions with oxygen-rich zirconium–organic coordination surfaces, particularly in nitrate-dominated systems.^24^ Nitrate anions can compete with uranyl ions for adsorption sites or alter the speciation of uranium in solution, affecting both the kinetics and thermodynamics of sorption.^25^ In addition, stability and selectivity in realistic matrices—where competing ions and variable acidity may be present—remain decisive criteria for implementation. Therefore, a comprehensive investigation into the adsorption behavior of U(vi) on zirconium-gallic acid coordination materials is essential. This includes evaluating the adsorption capacity under various pH conditions, determining kinetic and isotherm parameters, exploring thermodynamic properties, and elucidating the dominant binding mechanisms through spectroscopic and modeling techniques.^24–26^

In this contribution, a zirconium-gallic acid-based metal–organic compound (Zr-GA) is synthesized and investigated as an efficient adsorbent for U(vi) removal from nitrate-containing aqueous systems. The material is structurally and chemically characterized using XRD, FTIR, XPS, SEM-EDX, BET, and DLS techniques. Batch adsorption experiments are performed to assess the effect of pH, contact time, adsorbent dose, temperature, and initial concentration. Kinetic, isotherm, and thermodynamic models are applied to evaluate sorption behavior and elucidate the adsorption mechanism. Furthermore, regeneration and desorption studies are conducted to assess the reusability of the material, and a real radioactive effluent is tested to validate performance under practical conditions. This work offers new insights into the potential of Zr-GA as a robust and selective adsorbent for uranium remediation and nuclear wastewater treatment.

## 2. Experiments

### 2.1. Materials

All chemicals used in this study were of analytical grade and used without further purification. Zirconium(iv) chloride (ZrCl_4_, ≥99.9%) and gallic acid (C_7_H_6_O_5_, ≥98%) were obtained from Sigma-Aldrich (UK). *N*,*N*-Dimethylformamide (DMF, 99.5%) and ethanol (95%) were supplied by Sigma-Aldrich, while nitric acid (HNO_3_), hydrochloric acid (HCl), sulfuric acid (H_2_SO_4_), sodium hydroxide (NaOH), and sodium carbonate (Na_2_CO_3_) were purchased from Merck (Germany) and used as received. Uranium(vi) was introduced as uranyl nitrate hexahydrate (UO_2_(NO_3_)_2_·6H_2_O, Merck, ≥98%). All aqueous solutions were prepared using double-distilled water. Arsenazo III (Sigma-Aldrich) was used as a colorimetric reagent for the spectrophotometric determination of U(vi).

### 2.2. Synthesis of the adsorbent

The Zr-GA was synthesized *via* a one-step hydrothermal method, adapted with modifications from a previously reported procedure.^27^ In a typical synthesis, 3.0 mmol of ZrCl_4_ (0.666 g) was dissolved in 50 mL of a solvent mixture composed of distilled water and DMF in a 3 : 2 volume ratio. In parallel, 1.0 mmol of gallic acid (0.170 g) was dissolved in 50 mL of distilled water. The ZrCl_4_ solution was slowly added dropwise to the gallic acid solution under continuous magnetic stirring to ensure homogeneous mixing.

**Fig. 1:**
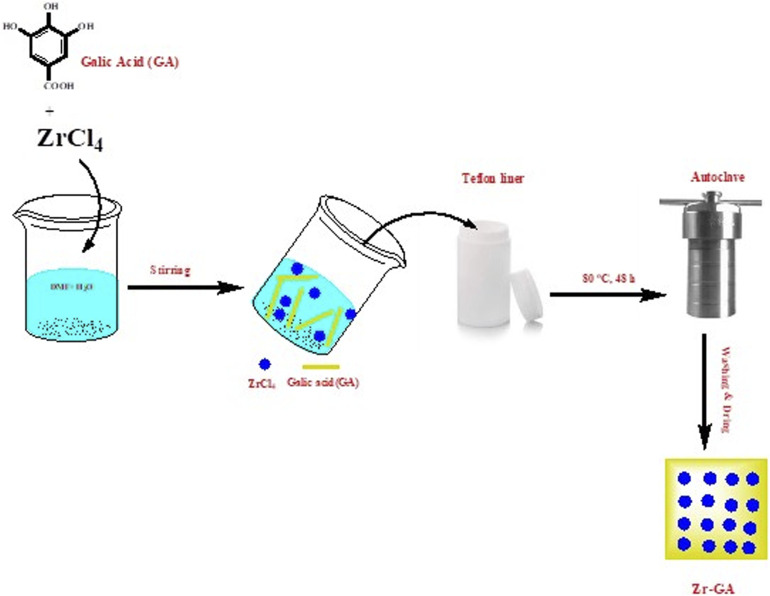
The synthetic pathway for the adsorbent preparation.

The resulting clear brown solution was transferred to a 100 mL Teflon-lined stainless-steel autoclave, sealed, and heated at 80 °C for 48 h in a programmable oven. After natural cooling to room temperature, a reddish-brown precipitate was recovered by vacuum filtration and washed thoroughly with ethanol and distilled water to remove unreacted precursors. The final product was dried under vacuum at 60 °C for 12 h and stored in a desiccator for further use. A schematic representation of the synthesis process is shown in Fig. 1.

### 2.3. Adsorbent characterization

The synthesized Zr-GA was characterized using several analytical techniques to confirm its structural, morphological, and chemical features. X-ray diffraction (XRD) analysis was performed using a PANalytical X'Pert PRO diffractometer (Netherlands) with Cu Kα radiation (*λ* = 1.5406 Å) operated at 40 kV and 30 mA, scanned over the 2*θ* range of 5–60°. Fourier-transform infrared spectroscopy (FTIR) spectra were recorded on a PerkinElmer Spectrum Two spectrometer in the 4000–400 cm^−1^ range using the KBr pellet method. Surface morphology and elemental distribution were examined using scanning electron microscopy (SEM) equipped with energy-dispersive X-ray spectroscopy (EDX) (JEOL JSM-6510LV, Japan). Transmission electron microscopy (TEM) was conducted using a JEOL JEM-2100 microscope at 200 kV to further analyze internal structure. Specific surface area, pore volume, and pore diameter were measured by nitrogen adsorption–desorption isotherms using the BET method (Quantachrome NOVA 2000e analyzer). Thermal stability was evaluated by thermogravimetric analysis (TGA, PerkinElmer TGA 4000) in a nitrogen atmosphere at a heating rate of 10 °C min^−1^. Zeta potential and hydrodynamic particle size were determined using a Malvern Zetasizer Nano ZS90. X-ray photoelectron spectroscopy (XPS) measurements were performed using a Thermo Scientific ESCALAB 250Xi system with Al Kα radiation to investigate elemental states and chemical environments.

### 2.4. Adsorption experiments

All batch adsorption experiments were conducted in 0.1 M nitrate medium to simulate typical aqueous environments encountered in uranium-bearing effluents. Investigations were performed in 100 mL polypropylene centrifuge tubes using a digital Thermo-shaker water bath (Model SWB 27, Scientific Precision, USA). A fixed volume of uranium(vi) solution (50 mL) was treated with a known dose of Zr-GA under controlled shaking conditions (150 rpm) at 25 ± 1 °C. After equilibrium, the suspensions were filtered through 0.22 µm syringe filters, and the residual U(vi) concentration was determined spectrophotometrically using A UV-Vis spectrophotometer (Lambda, PerkinElmer, USA) at 652 nm using Arsenazo III reagent. Cautionary note: Uranium compounds are radioactive and chemically toxic. All experiments involving U(vi) were conducted using dilute aqueous solutions under controlled laboratory conditions in accordance with institutional radiation safety procedures. Appropriate PPE (lab coat, gloves, and safety glasses) was used, and uranium-containing solutions/solids were collected and disposed of through approved radiological and chemical waste management routes.

The effects of pH (1–6), contact time (3–240 min), adsorbent dosage (0.4–1.5 g L^−1^), initial U(vi) concentration (20–300 mg L^−1^), and temperature (298–328 K) were systematically investigated. The initial solution pH was adjusted using 0.1 M HCl or NaOH. Each experiment was repeated in triplicate, and average values with deviations below 5% were accepted. The amount of U(vi) adsorbed at equilibrium (*q*_e_, mg g^−1^), removal efficiency (E%), and distribution coefficient (*K*_d_, L g^−1^) were calculated using the following expressions:1
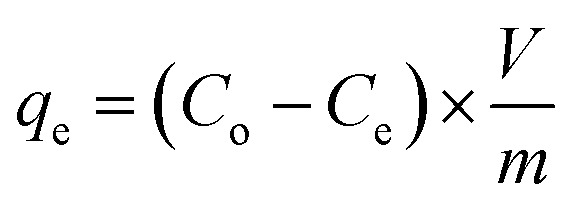
2
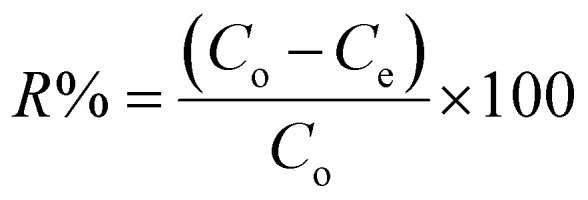
3
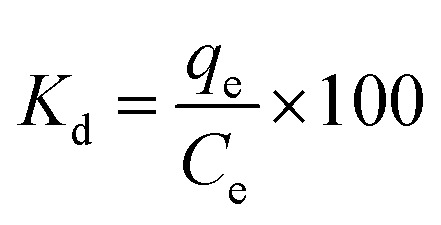
where *C*_o_ and *C*_e_ are the initial and equilibrium U(vi) concentrations (mg L^−1^), *V* is the volume of solution (L), and *m* is the mass of adsorbent (g).

To elucidate the adsorption mechanism and quantify adsorption behavior, kinetic, isotherm, and thermodynamic analyses were conducted. All applied models and their mathematical expressions are provided in Table S1. The kinetic data were fitted to the pseudo-first-order, pseudo-second-order, Elovich, and intraparticle diffusion (Weber–Morris) models to determine the rate-controlling steps and nature of the adsorption process. Additionally, Boyd's diffusion model was applied to distinguish between external (film) and internal (pore) diffusion control. Model parameters were obtained using non-linear regression, and fits were assessed based on the coefficient of determination (*R*^2^) and average relative error (ARE). Equilibrium data were analyzed using the Langmuir, Freundlich, and Sips isotherm models to evaluate surface homogeneity, maximum adsorption capacity, and binding affinity. The best-fitting model was selected based on statistical criteria, including *R*^2^ and error minimization. The dimensionless separation factor (*R*_L_) was also calculated from the Langmuir constant to assess the favorability of adsorption. Thermodynamic parameters—Gibbs free energy (Δ*G*°), enthalpy change (Δ*H*°), and entropy change (Δ*S*°)—were evaluated using the van't Hoff equation, where the base-10 logarithm of the equilibrium constant (log *K*_c_) was plotted against the reciprocal of temperature (1/T). The equations used are detailed in Table S1, and values were derived from equilibrium data collected at 298, 308, 318, and 328 K. These models were applied following established procedures reported in the literature,^28–35^ and were used to derive mechanistic insights regarding the interaction of U(vi) with the Zr-GA surface.

Desorption experiments were performed to assess the regeneration efficiency of Zr-GA after uranium uptake. Several eluents were tested, including 1.0 M solutions of HCl, HNO_3_, H_2_SO_4_, and Na_2_CO_3_. The uranium-loaded adsorbent was treated with 50 mL of each eluent under agitation for 4 h, and the concentration of uranium in the eluent was determined to calculate desorption efficiency. Based on the results, the best-performing eluent was selected for five-cycle reuse testing. The practical applicability of the Zr-GA was assessed using a liquid waste sample obtained from the Nuclear Materials Authority of Egypt. The solution contained U(vi) (100 mg L^−1^), along with high levels of Fe(iii) and Ca(ii), in a 0.5 M nitric acid matrix. The adsorbent was tested under optimized conditions (pH 3.01, contact time 120 min, dose 1 g L^−1^), and the U(vi) removal efficiency was evaluated to determine the material's selectivity and performance in complex systems.

## 3. Results & discussion

### 3.1. Characterizations of the adsorbent

A series of complementary analytical techniques were employed to elucidate the structural, morphological, surface, and chemical properties of the synthesized Zr-GA. These analyses collectively confirm the successful formation of a stable and functional sorbent suitable for uranium adsorption.

#### 3.1.1. XRD analysis

**Fig. 2:**
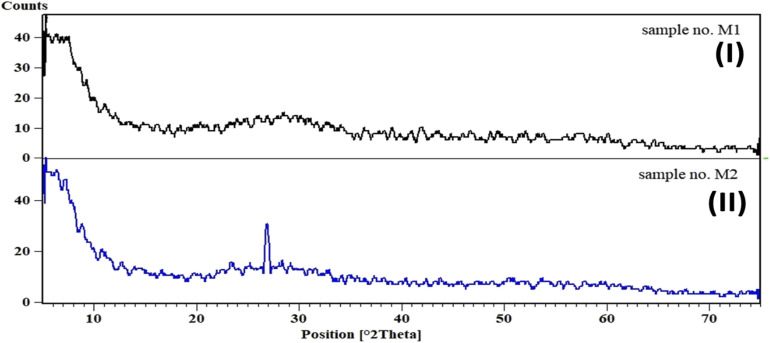
XRD pattern of the gallic acid (I), and the Zr-GA (II) adsorbent.

The X-ray Diffraction (XRD) analysis of gallic acid (GA) and the Zr-Gallic acid material (Zr-GA) is presented in Fig. 2. The XRD pattern of GA (Fig. 2I) displays a broad signal between 10° and 30°, which is characteristic of amorphous organic compounds and reflects the absence of long-range crystallinity, consistent with the known structural nature of gallic acid.^36^ In contrast, the XRD pattern of the Zr-GA material (Fig. 2II) exhibits a dominant reflection centered at approximately 27° (2*θ*), accompanied by a broad diffraction background in the 20–33° region, indicating partial structural ordering rather than extensive long-range periodicity. Accordingly, the XRD data are interpreted as supportive “fingerprint” evidence of coordination-induced structural organization, and not as standalone proof of a fully crystalline structure or phase purity. While the observed diffraction feature is consistent with the formation of zirconium–oxygen–carboxylate/phenolate coordination linkages, no specific crystallographic topology is assigned, as a validated CIF file for a zirconium-gallic acid organic compound is not available and the product forms as fine domains unsuitable for single-crystal XRD analysis. Moreover, although the synthetic strategy was adapted from a coordination-based approach reported for Fe(iii)–gallic systems, the coordination chemistry of Zr(iv) differs fundamentally from that of Fe(iii); therefore, structural features reported for Fe–gallic materials cannot be directly extrapolated to the present Zr-based system.^15–17^

The XRD sample was prepared by gentle grinding of the dried powder followed by mounting on a low-background holder. The relatively elevated background intensity and modest diffraction signal are attributed to the organic-rich composition of the material, limited crystallite domain size, and the absence of long-range periodic order, features commonly observed for metal–polyphenol coordination solids.^15–17^ It is also clarified that the pattern shown in Fig. 2II corresponds to the experimental diffraction profile of the synthesized Zr-GA material rather than a database reference or simulated pattern. Since no JCPDS card exists for this system, the present XRD data serve only as experimental evidence of coordination-induced phase formation. The overall diffraction envelope differs from that of zirconium precursor salts (*e.g.*, ZrOCl_2_·8H_2_O), supporting the formation of a new zirconium–organic coordination solid rather than residual inorganic precursor.^15–17^

Importantly, XRD alone does not establish phase purity or definitive crystallinity for the Zr-GA material. Instead, confirmation of successful material formation and functional integrity is derived from combined characterization, including FTIR evidence of coordinated carboxylate groups, XPS confirmation of Zr–O–C bonding environments, SEM–EDS elemental homogeneity, and consistent adsorption behavior. Thus, PXRD is used here as a complementary technique to support coordination-induced structural organization, without over-interpretation of crystallinity or phase purity.

#### 3.1.2. FTIR analysis

**Fig. 3:**
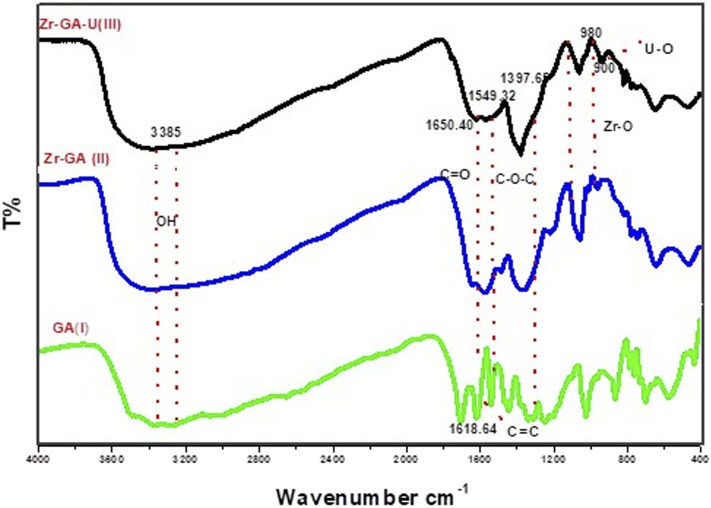
FTIR analysis of the gallic acid (I), Zr-GA before uranium adsorption (II), and after adsorption process (III).

The FTIR spectra of gallic acid (GA), Zr-GA before adsorption, and uranium-loaded Zr-GA are shown in (Fig. 3). These spectra provide insights into the structural changes occurring upon the coordination of GA with Zr(iv) and subsequent uranium adsorption. The spectrum of GA (Fig. 3I) displays a broad absorption band at 3386.71 cm^−1^ (O–H stretching), confirming the presence of hydroxyl groups.^36,37^ The distinct peak at 1685.51 cm^−1^ (C=O stretching of –COOH) is attributed to the carboxylic acid group, while the band at 1618.64 cm^−1^ (aromatic C=C stretching) reflects aromatic skeletal vibrations. The region between 1500 and 1000 cm^−1^ includes bands for C–O stretching and aromatic skeletal vibrations, and signals below 1000 cm^−1^ correspond to out-of-plane bending modes characteristic of phenolic and carboxylic functionalities.^36–38^ For clarity, the main bands and their assignments are now labeled directly in (Fig. 3).

Following formation of the Zr-GA, the FTIR spectrum (Fig. 3II) shows several key shifts and new features. The O–H stretching band remains broad at 3385.57 cm^−1^, which may reflect residual water and/or coordinated hydroxyl groups. The band assigned to carboxylic C=O at 1685.51 cm^−1^ decreases/changes and is accompanied by carboxylate features, while peaks at 1549.32 cm^−1^ and 1397.65 cm^−1^ correspond to the asymmetric and symmetric stretching vibrations of COO^−^, supporting deprotonation and coordination of GA to Zr(iv).^38^ These coordination-induced shifts are interpreted as evidence of ligand binding through oxygen-donor groups; however, FTIR does not resolve a definitive coordination geometry. In the fingerprint region, bands below 1000 cm^−1^ are consistent with metal–oxygen (Zr–O) vibrations, supporting zirconium–oxygen linkage formation in the Zr-GA structure.^38^

Upon U(vi) adsorption, the FTIR spectrum of the loaded Zr-GA (Fig. 3III) exhibits further modifications. The O–H stretching band shifts slightly to 3385.95 cm^−1^, indicating altered hydrogen bonding and/or interaction of uranyl species with surface hydroxyl groups. A band at 1728.48 cm^−1^ may correspond to residual non-coordinated –COOH and/or adsorption-induced perturbation of carbonyl environments.^36,37^ The COO^−^ bands at 1650.84 cm^−1^ and 1398.38 cm^−1^ show slight shifts and intensity changes, consistent with the participation of carboxylate/phenolic oxygen sites in U(vi) binding. Any assignment of uranyl binding is therefore made on the basis of these FTIR changes together with complementary XPS evidence and adsorption modeling, rather than FTIR alone.^36,37^ All key bands (3386, 1686, 1619, 1549, 1398, and <1000 cm^−1^) are now annotated in (Fig. 3) to link the spectra to functional-group assignments.

#### 3.1.3. XPS analyses

**Fig. 4:**
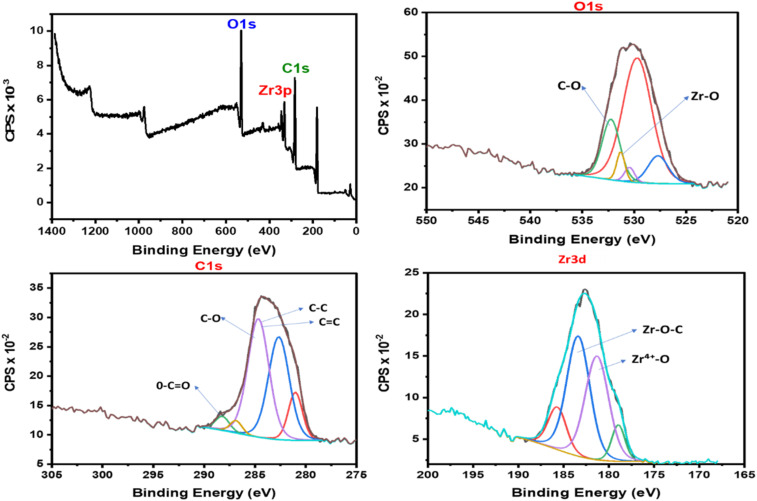
XPS analyses of the applied Zr-GA adsorbent.

X-ray photoelectron spectroscopy (XPS) was employed to investigate the surface chemical composition and elemental states of the Zr-GA (Fig. 4). Prior to analysis, the powdered samples were dried under vacuum, gently pressed onto conductive carbon tape, and analyzed without further chemical treatment. The wide-scan survey spectrum reveals distinct peaks corresponding to C 1s, O 1s, and Zr 3p, confirming the presence of carbon, oxygen, and zirconium as the primary elements in the Zr-GA structure. Quantitative analysis showed a surface atomic composition of 70.09% carbon, 25.65% oxygen, and 4.26% zirconium. This distribution is consistent with the expected structure of a zirconium-gallic acid metal–organic compound, contributing extensively to the carbon content, while zirconium acts as the inorganic coordination center.^39,40^

The high-resolution C 1s spectrum (Fig. 4a) was deconvoluted into three main peaks: 284.8 eV attributed to C–C/C=C from aromatic rings, 286.2 eV to C–O from phenolic groups, and 288.5 eV to O–C=O from carboxylate species. Similarly, the O 1s spectrum (Fig. 4b) is discussed in terms of two dominant contributions, located at 531.6 eV and 533.2 eV, which are attributed to metal–oxygen (Zr-O) and C–O/–OH environments, respectively. Higher-energy shoulders present in the envelope were not assigned individually, as they likely arise from overlapping oxygen environments common in organic-rich coordination materials.

The Zr 3d region (Fig. 4c) displays a characteristic doublet with binding energies at 182.2 eV (Zr 3d_5/2_) and 183.1 eV (Zr 3d_3/2_), confirming the presence of Zr^4+^ in an oxygen-coordinated environment. All high-resolution spectra were fitted using a Shirley background and mixed Gaussian–Lorentzian (GL) line shapes, with constraints applied to peak positions and full width at half maximum (FWHM) to ensure chemically reasonable fitting.^39^ Overall, the XPS results provide supportive evidence for zirconium–oxygen coordination with gallic acid functional groups, in agreement with FTIR analysis. XPS is used here to confirm surface composition and chemical-state consistency, rather than to resolve a detailed coordination geometry. The presence of oxygen-rich functional groups (phenolic and carboxylate) provides abundant active sites for uranium(vi) adsorption *via* inner-sphere complexation and electrostatic interaction.^40^

#### 3.1.4. BET analyses

The Brunauer–Emmett–Teller (BET) analysis of the Zr-GA provides essential information about its textural properties, which are directly linked to its adsorption performance (Table 1). The material exhibited a specific surface area of 37.9 m^2^ g^−1^, reflecting a moderate accessible surface area for an organic-rich coordination solid. Although this value is lower than that reported for many ultra-porous sorbents, it remains within the expected range for zirconium–organic coordination materials designed for selective binding rather than maximized surface area.^36,37^ The measured total pore volume was 6.01 × 10^−2^ cm^3^ g^−1^, and the average pore diameter was ∼3.2 nm. While this pore size falls within the mesopore range (2–50 nm) according to IUPAC classification, it is most reasonably attributed to textural porosity arising from interparticle voids and particle packing effects, rather than intrinsic structural mesopores.^37–39^

**Table 1:** Surface properties of the applied Zr-GA adsorbent

Total pore volume, cm^3^ g^−1^	Average pore size, nm	Surface area (m^2^ g^−1^)
6.0058 e−002	3.1657	37.943

Accordingly, the BET results are interpreted as evidence of accessible surface area and favorable textural characteristics that facilitate diffusion and mass transfer during adsorption, rather than proof of a permanently mesoporous metal–organic compound architecture. These textural features can support the uptake of metal ions such as U(vi) by providing short diffusion pathways and readily accessible oxygen-donor functional sites. The combination of moderate surface area with mesoporosity likely reflects an optimized balance between structural integrity and targeted adsorption behavior. Rather than relying on surface area alone, the Zr-GA appears designed to promote selective binding interactions—an essential feature in uranium recovery applications. The presence of interparticle mesopore-range voids may also contribute to efficient transport and favorable kinetics, supporting adsorption performance even at elevated concentrations.^36–38^

#### 3.1.5. Zeta potential and DLS analyses

The Zr-GA exhibited a highly negative average zeta potential of −46.69 mV, as presented in Table S2, indicating a strongly negative surface charge that generates electrostatic repulsion between particles and minimizes aggregation in aqueous suspension. This dispersion behavior is critical in adsorption applications, as it ensures high dispersion and maximum surface accessibility for interaction with uranium ions. Zeta potential measurements were performed in deionized water, consistent with the instrument parameters (viscosity ≈ 0.995 cP; refractive index 1.333; dielectric constant 78.5). After uranium adsorption, the zeta potential decreased markedly to −26.49 mV, reflecting partial neutralization of the Zr-GA surface charge by the positively charged U(vi) species. This reduction confirms the electrostatic interaction and binding of uranium onto the negatively charged surface groups of the sorbent, which modifies its surface chemistry and could increase its tendency toward agglomeration due to diminished repulsive forces.^41,42^

**Fig. 5:**
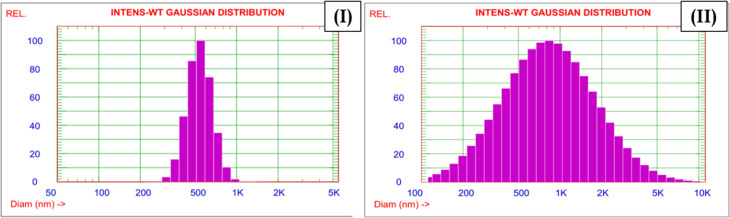
Particle size distribution of Zr-GA before (I), and after (II) adsorption process.

Dynamic Light Scattering (DLS) was employed to evaluate changes in particle size distribution before and after uranium adsorption (Fig. 5, Table S2). Prior to adsorption, the Zr-GA displayed a narrow size distribution centered at 549.5 nm, with a polydispersity index (PDI) of 0.045, indicating a relatively uniform and well-dispersed suspension under the measurement conditions. Following uranium uptake, the average particle size nearly doubled and the distribution broadened significantly. This shift implies strong interaction between uranium ions and the Zr-GA's surface functional groups, potentially leading to particle aggregation or structural expansion due to surface complexation.^41–43^ These observations validate the strong affinity of Zr-GA toward uranium ions and highlight its potential application in nuclear waste remediation. The substantial changes in both surface charge and hydrodynamic particle size reflect key physicochemical interactions associated with the adsorption process, supporting a mechanism dominated by surface complexation and electrostatic attraction.^38^

#### 3.1.6. SEM and EDX analysis

**Fig. 6:**
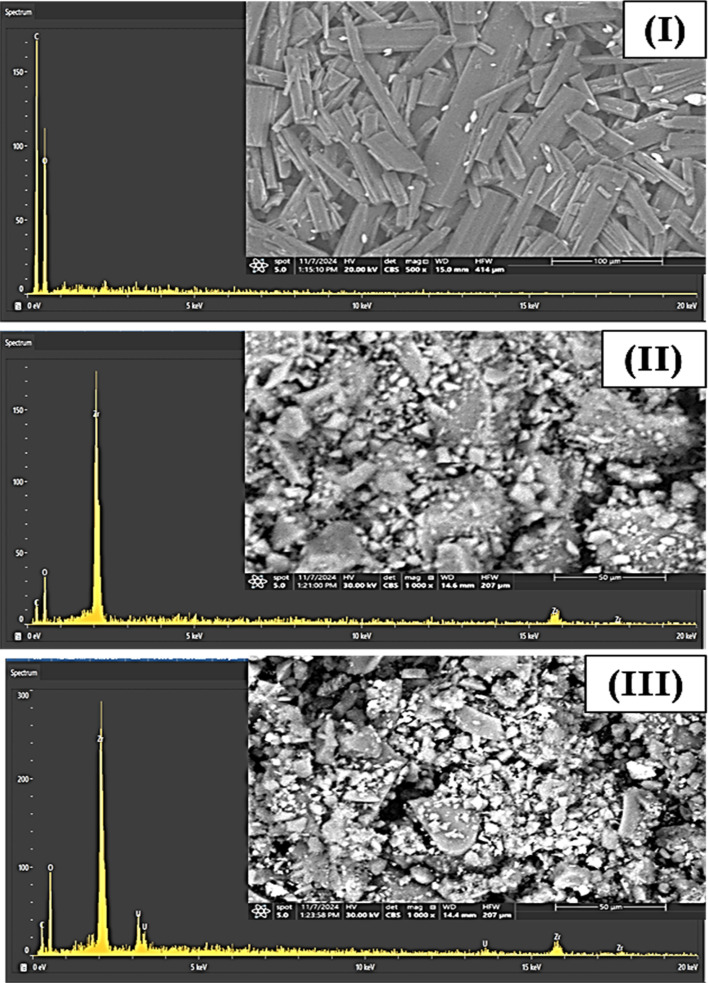
SEM and EDS analysis of the gallic acid (I), Zr-GA before uranium adsorption (II), and after adsorption process (III).

Scanning Electron Microscopy (SEM) coupled with Energy-Dispersive X-ray Spectroscopy (EDS) was employed to examine the morphological features and elemental composition of the gallic acid precursor, the synthesized Zr-GA, and the U(vi)-loaded sorbent (Fig. 6). The SEM/EDS image of gallic acid (Fig. 6I) is included solely for qualitative comparison and does not form the basis for any structural or mechanistic interpretation; accordingly, the discussion focuses primarily on the Zr-GA before and after U(vi) adsorption. The SEM micrographs of the pristine Zr-GA (Fig. 6II) reveal aggregated micro-to sub-micrometric particles with irregular shapes and relatively rough surface textures. These observations provide qualitative insight into particle morphology and aggregation behavior but do not convey information about the zirconium coordination environment, which is instead elucidated through spectroscopic techniques such as FTIR and XPS (Sections 3.1.2 and 3.1.3). The observed morphology is typical of zirconium-based metal–organic compound synthesized *via* solution routes and is consistent with a uniform material formation process.^36–38^ Following U(vi) adsorption, the SEM image of the U-loaded Zr-GA (Fig. 6III) shows moderate surface roughening and increased compactness of particle aggregates. Since adsorption experiments were conducted under acidic conditions (pH ≈ 3.0), these morphological changes are attributed to a combination of acidic exposure and uranium uptake rather than being interpreted as direct evidence of specific coordination geometry. Accordingly, SEM observations are discussed conservatively as indicators of surface modification following treatment.^37,38^

All SEM samples were prepared by mounting the dry powders on conductive carbon tape and sputter-coating with a thin gold layer prior to imaging in order to minimize charging effects. Some residual charging is nevertheless observed, which is common for organic-rich materials and does not affect the qualitative morphological assessment. It is also important to clarify that particle sizes observed by SEM are larger than those measured by Dynamic Light Scattering (DLS). This discrepancy arises because SEM images represent the dry, aggregated state of the particles, whereas DLS measures the hydrodynamic diameter of dispersed particles in aqueous suspension. The smaller particle sizes obtained from DLS therefore reflect dispersion behavior under solution conditions relevant to adsorption experiments rather than the dry morphology observed by SEM.^41–43^

Complementary to the morphological observations, EDS analysis confirms the elemental composition of the samples. In the pristine Zr-GA, distinct peaks for Zr, C, and O are present, aligning with the expected formulation. The uniform spatial distribution of these elements indicates homogeneous integration of organic and inorganic components. Following uranium adsorption, new peaks appear in the EDS spectrum corresponding to U, verifying its successful uptake. The consistent intensity and distribution of uranium signals across the surface confirm uniform adsorption, highlighting the high affinity and capacity of the Zr-GA for U(vi) ions.^36–38^

In conclusion, the comprehensive characterization demonstrates the successful synthesis of a structurally robust, crystalline, and functionally active Zr-GA. High crystallinity and purity were confirmed by XRD; FTIR and XPS verified functional group coordination; SEM/EDX revealed particle morphology and elemental distributions (including U after loading), while DLS captured changes in hydrodynamic size; zeta potential indicated a strongly negative surface charge and good dispersion behavior in aqueous suspension; and N_2_ adsorption–desorption (BET/BJH) data confirmed the mesoporous character (type IV isotherm with H_3_ hysteresis) and accessible diffusion channels. Together, these findings underscore the material's potential for highly selective and efficient uranium(vi) adsorption from aqueous environments.

### 3.2. Batch investigation

**Fig. 7:**
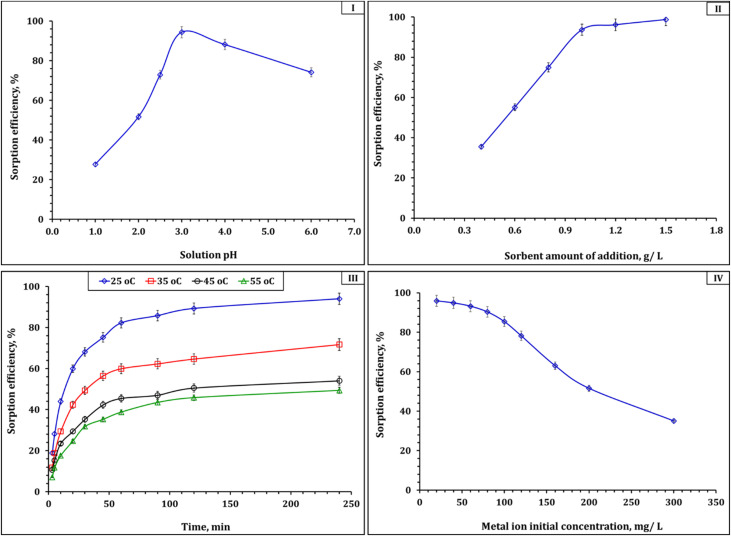
Impact of various conditions on U(vi) adsorption efficiency (I) solution pH: adsorbent dose 1.0 g L^−1^, time 240 min, initial concentration of 50 mg L^−1^, 25 °C; (II) shaking time: solution pH of 3.01, adsorbent dose 1.0 g L^−1^, initial concentration of 50 mg L^−1^, 25 °C; (III) adsorbent dose: solution pH of 3.01, time 240 min, initial concentration of 50 mg L^−1^, 25 °C; (IV) initial concentration: solution pH of 3.01, time 240 min, adsorbent dose 1.0 g L^−1^, 25 °C.

To optimize the adsorption performance of the synthesized Zr-GA for uranium removal from aqueous solutions, the effects of several key operating parameters—namely solution pH, adsorbent dosage, contact time, temperature, and initial uranium concentration—were systematically investigated. These variables directly influence the surface charge, uranium speciation, active site availability, and interaction kinetics between the adsorbent and U(vi) ions. All experiments were conducted in 0.1 M nitric acid, a relevant condition for modeling uranium behavior in liquid waste solutions. The effects of these parameters on adsorption efficiency are summarized in Fig. 7.

The pH of the solution plays a particularly important role in influencing both uranium speciation and the surface charge of the Zr-GA, thereby governing the adsorption mechanism. As shown in Fig. 7I, the U(vi) removal efficiency was highly pH-dependent, increasing significantly from ∼35% at pH 1.0 to a maximum of 94.4% at pH 3.0, before declining at higher pH values. Speciation modeling using Medusa–Hydra software (Fig. S1) indicates that UO_2_^2+^ and UO_2_NO_3_^+^ are the dominant species below pH 4, where electrostatic attraction with the negatively charged sorbent surface is most favorable.^44^ This behavior can be attributed to the strong electrostatic interaction between the negatively charged surface (zeta potential = −46.69 mV) and positively charged uranyl species under mildly acidic conditions.^45,46^ At pH 3.0, uranyl ions remain predominantly in cationic form and interact effectively with deprotonated –OH and –COOH groups on the adsorbent, promoting inner-sphere complexation.^45^ At pH values below 2.0, competitive protonation of surface sites reduces binding affinity. Above pH 4.0, the emergence of neutral or anionic uranyl hydroxide species such as (UO_2_)_2_(OH)_2_^2+^, (UO_2_)_3_(OH)_5_^+^, and UO_2_(OH)_4_^2−^ leads to reduced electrostatic attraction and lower uptake efficiency.^44–46^ Moreover, the onset of precipitation as UO_2_(OH)_2_·H_2_O(s) at higher pH values further limits soluble uranium availability.^45,46^

Adsorbent dosage also exerted a significant impact on removal efficiency. As the dose of Zr-GA increased from 0.4 to 1.5 g L^−1^, uranium removal improved from 35.5% to 98.7% (Fig. 7II). This improvement is directly attributed to the increased number of active binding sites, which allows for more complete capture of U(vi) ions.^45,46^ This trend is consistent with the material's morphological and surface characteristics described earlier, including the mesoporous structure (∼3.17 nm), moderate BET surface area (37.9 m^2^ g^−1^), and uniformly distributed surface functionalities (Sections 3.1.3–3.1.5). At lower dosages, limited functional groups become saturated quickly. With increasing dosage, more adsorption sites become available, resulting in more efficient uranium uptake. However, beyond 1.0 g L^−1^, the increase begins to plateau, reflecting saturation behavior and a common limitation in batch adsorption systems, where excess adsorbent does not proportionally enhance removal.^45–47^

The effect of contact time was assessed simultaneously with the impact of temperature to evaluate both kinetic and thermodynamic behavior (Fig. 7III). At 25 °C, rapid uptake was observed during the first 60 min (82.3% removal), followed by a more gradual increase to 94.0% by 240 min. This biphasic profile is characteristic of initial fast adsorption on accessible surface sites, followed by slower intraparticle diffusion into mesopores.^47–49^ The efficiency of the process is further supported by the presence of carboxyl and hydroxyl groups (confirmed by FTIR and XPS), and the mesoporous architecture described by BET analysis. These features facilitate short diffusion paths and strong metal–ligand coordination, enhancing uptake kinetics.

Interestingly, increasing temperature had an inverse effect on adsorption. Maximum removal efficiencies at 240 min dropped to 71.7%, 54.1%, and 49.4% at 35, 45, and 55 °C, respectively. These results confirm that the process is exothermic, with higher temperatures weakening coordination bonds between U(vi) and the MOF's oxygen donor sites.^28,29^ Thermal energy may also destabilize the uranyl complexation environment or promote competing hydrolysis pathways, both of which reduce binding affinity.^28,29^ These findings highlight the advantage of ambient temperature operation, which is favorable for both performance and energy efficiency in environmental applications.

Initial uranium concentration also played a major role in determining removal efficiency. As the U(vi) concentration increased from 20 to 300 mg L^−1^, the removal percentage decreased from 95.9% to 35.0% (Fig. 7IV). This trend is attributed to the fixed number of adsorption sites on the sorbent in a batch system. At low concentrations, excess active sites result in nearly complete uptake. As the concentration increases, site saturation occurs more quickly, leaving more U(vi) unadsorbed in solution.^45,46^ Notably, the Zr-GA maintained high efficiency (>85%) up to 100 mg L^−1^ and still retained over 50% removal at 200 mg L^−1^, demonstrating strong affinity and significant capacity under relevant loading scenarios.^45,46^

Taken together, these findings provide a comprehensive understanding of the operational parameters governing uranium adsorption onto Zr-GA. Optimal adsorption occurs at pH 3.0, with an adsorbent dosage of ∼1.2 g L^−1^, an equilibrium time of 240 min, and under ambient temperatures. The sorbent maintains respectable performance across a range of uranium concentrations, supporting its application in diverse water treatment scenarios. These parameters not only reflect the material's intrinsic affinity for uranium but also highlight its practical robustness and adaptability under real-world environmental conditions. Importantly, these trends correlate strongly with the physicochemical insights obtained from adsorbent characterization, particularly the abundance of oxygen donor groups, high negative surface charge, and accessible mesoporosity that together enable efficient U(vi) capture.

The batch investigation also underscores the practical applicability of the synthesized Zr-GA as an effective ligand for capturing metallic pollutants. Its adsorption performance is influenced by pH, initial ion concentration, temperature, contact time, and dose, all of which interact with the material's structural and surface properties. Comparable trends have been reported for other uranium adsorbents such as amino-functionalised cross-linked polyacrylamide,^45^ polyamide-based nanocomposites,^46^ and phosphorylated luffa rattan activated carbon,^50^ further validating the significance of these findings for real-world applications in wastewater treatment and environmental remediation.

### 3.3. Adsorption kinetic investigation

The time-dependent adsorption behavior of U(vi) onto the synthesized Zr-GA was evaluated using several kinetic models to elucidate the underlying adsorption mechanism and rate-controlling steps. These models included the pseudo-first-order (PFO) and pseudo-second-order (PSO) models, as well as intraparticle diffusion (Weber–Morris), the Boyd film diffusion model, and an Arrhenius analysis based on diffusion coefficients. The kinetic profile revealed a two-stage process: a rapid uptake phase followed by a slower approach toward equilibrium. This biphasic behavior reflects the initial abundance of active binding sites on the sorbent surface, which gradually become saturated, leading to diffusional constraints.^28–31^ The sharp initial slope is consistent with fast surface interactions—likely chemisorption—on oxygen-rich functional groups such as carboxylates and phenols, as confirmed by FTIR and XPS (Sections 3.1.2 and 3.1.6). Equilibrium was reached after approximately 240 min, which aligns well with the contact time optimization discussed in Section 3.2.

**Fig. 8:**
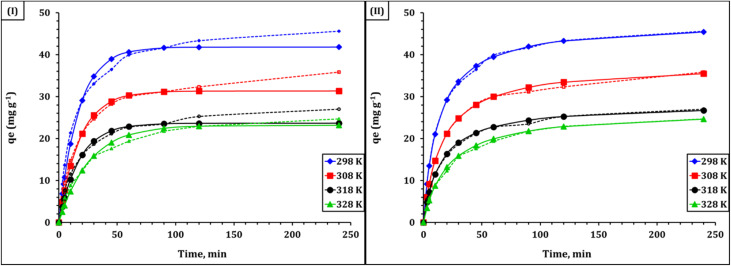
Kinetic modeling of U(vi) adsorption onto Zr-GA: (I) pseudo-first-order model and (II) pseudo-second-order model.

For kinetic modeling, the pseudo-first-order rate constant (*k*_1_) and equilibrium capacity (*q*_e_) were obtained from the linear plot of log (*q*_e_ – *q*_*t*_) *versus* time (*t*), while the pseudo-second-order rate constant (*k*_2_) and *q*_e_ were determined from the plot of t/*q*_*t*_*versus t* (Fig. 8I and II). As shown in Fig. 8(I and II) and summarized in Table 2, the Lagergren pseudo-first-order model Fig. 8(I), which assumes that the rate of adsorption is proportional to the number of unoccupied sites,^28,29^ provided a reasonable but not ideal fit to the experimental data. The calculated equilibrium capacity (*q*_1_) was 48.92 mg g^−1^, with a rate constant (*k*_1_) of 0.0182 min^−1^ and a correlation coefficient (*R*^2^) of 0.925. The relatively high average relative error (ARE = 5.35) indicates a modest deviation between calculated and experimental values, particularly during the later stages of the process. In contrast, the PSO model Fig. 8 (II)—based on the assumption that chemisorption is the rate-limiting step involving valence forces through electron sharing or exchange^30,31^ demonstrated excellent agreement. The calculated *q*_2_ value (51.34 mg g^−1^) closely matched the experimental value, with a rate constant (*k*_2_) of 4.6 × 10^−4^ g mg^−1^ min^−1^, an *R*^2^ value of 0.999, and a low ARE of 1.21 (Table 2). In addition, the parameters *h* and *t*_1/2_ in Table 2 correspond to the initial adsorption rate (*h* = *k*_2_*q*_e_^2^) and the half-adsorption time (*t*_1/2_ = 1/*k*_2_*q*_e_), respectively, which further describe the kinetic behavior of the pseudo-second-order model.^30,31^ This strong agreement confirms the predominance of chemisorption in the uranium uptake mechanism. These results are fully consistent with the adsorbent's physicochemical features: a high density of –OH and –COOH groups (Sections 3.1.2 and 3.1.6), a highly negative surface potential (−46.69 mV, Section 3.1.4), and a mesoporous architecture (3.17 nm average pore size, section 3.1.5)—all of which promote UO_2_^2+^ interaction and retention. The same kinetic profile has been reported for uranium(vi) adsorption from aqueous solution using amino-functionalised cross-linked polyacrylamide,^45^ polyamide-based nanocomposites,^46^ and phosphorylated luffa rattan activated carbon.^50^

**Table 2:** Evaluated kinetic parameters based on pseudo-first-order and pseudo-second-order models

	25 °C	35 °C	45 °C	55 °C
**Pseudo first-order model**				

*q*_1_ (mg g^−1^)	41.80	31.34	23.64	23.14
*k*_1_ (min^− 1^)	0.059	0.056	0.057	0.038
*R* ^2^	0.98	0.98	0.97	0.98
**Pseudo second-order model**				

*q*_2_ (mg g^−1^)	47.8	37.8	28.3	26.8
*k*_2_ (min^−1^)	0.0024	0.0018	0.0017	0.0016
h (mol g^−1^ h^−1^)	3.8	2.4	1.9	1.3
*t*_1/2_ (h)	12.7	15.8	14.7	20.6
*R* ^2^	0.99	0.99	0.99	0.99

**Fig. 9:**
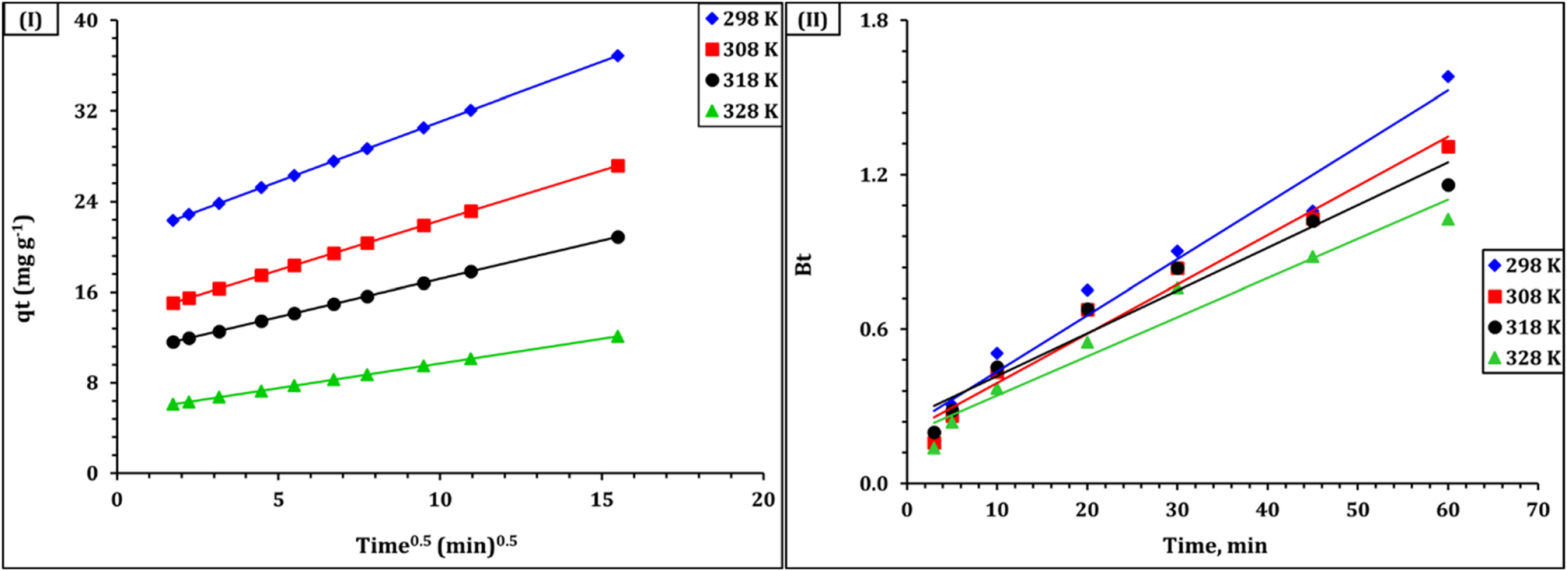
IPD (I), and Boyd (II) model plots for U(vi) diffusion into Zr-GA.

To further examine the rate-limiting mechanisms, the Weber–Morris intraparticle diffusion model was applied.^28,29^ As shown in Fig. 9I, the *q*_*t*_*vs. t*^1/2^ plots exhibited two distinct linear regions at all tested temperatures (298–328 K). The initial region corresponds to external film diffusion, while the second reflects intraparticle diffusion. The presence of multilinearity and non-zero intercepts confirms that intraparticle diffusion is involved but not the sole rate-determining step. Model parameters, including the slope (kᵢ), intercept (C), and correlation coefficient, are listed in Table S3. Notably, the decreasing slope with increasing temperature suggests reduced diffusion efficiency, supporting the exothermic nature of the process (see Section 3.2). To further distinguish between film and pore diffusion, the Boyd model was used.^28–31^ As illustrated in Fig. 9(II), the Bt *vs.* time plots were nonlinear and did not pass through the origin, confirming that film diffusion is the dominant mechanism during the initial adsorption stage. The steeper slopes observed at 298 K and 308 K indicate faster adsorption under ambient conditions, while the shallower slopes at elevated temperatures reinforce the conclusion that higher temperatures hinder uranium uptake. The Boyd-derived kinetic parameters are summarized in Table 3.

**Table 3:** Diffusion and activation energy values for the adsorption of U(vi) onto composite

Temp (K)	*D*_r_ × 10^−12^ (m^2^ S^−1^)	*D*_o_×X 10^−12^ (m^2^ S^−1^)	Ea (*k*_J_ mg^−1^)
298	1.67	0.029	−9.976
308	1.47		
318	1.27		
328	1.16		

These kinetic observations are in strong agreement with the textural porosity and chemically active surface of the adsorbent. The presence of abundant oxygen-containing functional groups (Sections 3.1.2 and 3.1.6), combined with mesopore-range textural voids arising from interparticle spacing (Section 3.1.5), supports a dual-stage kinetic process involving rapid surface interaction followed by diffusion-controlled mass transfer within the particle aggregates. Such behavior is commonly observed for metal–organic coordination materials and hybrid adsorbents, highlighting the combined roles of surface chemistry and pore accessibility in governing adsorption dynamics.

To assess the thermal effect on intraparticle diffusion, an Arrhenius analysis was performed using diffusion coefficients (Dᵣ) calculated from the linear portion of the Boyd plots. As shown in Fig. S2, a plot of ln Dᵣ *versus* 1/T yielded a straight line with a slope corresponding to an activation energy (*E*_a_) of 29.51 kJ mol^−1^, and a pre-exponential factor ln *D*_0_ of −19.72. These values are typical of thermally activated diffusion in mesoporous, chemisorptive systems.^45,46^ The moderate *E*_a_ confirms that diffusion through the sorbent channels encounters a measurable energy barrier, consistent with partial restriction due to pore geometry or internal surface interactions. The magnitude of Dᵣ, ranging from 10^−11^ to 10^−12^ m^2^ s^−1^, further supports the relevance of internal diffusion as a key contributor to the overall adsorption kinetics.^45,46^

In summary, the kinetic modeling results confirm that U(vi) adsorption onto Zr-GA is governed by a chemisorption-dominated mechanism, as best described by the pseudo-second-order model. The process is significantly influenced by dual diffusion regimes—initial film diffusion and subsequent intraparticle transport. The integration of kinetic, structural, and thermal analyses highlights the adsorbent's tailored efficiency for uranium capture under ambient conditions, reinforcing its suitability for practical environmental remediation applications.

### 3.4. Adsorption isotherms

**Fig. 10:**
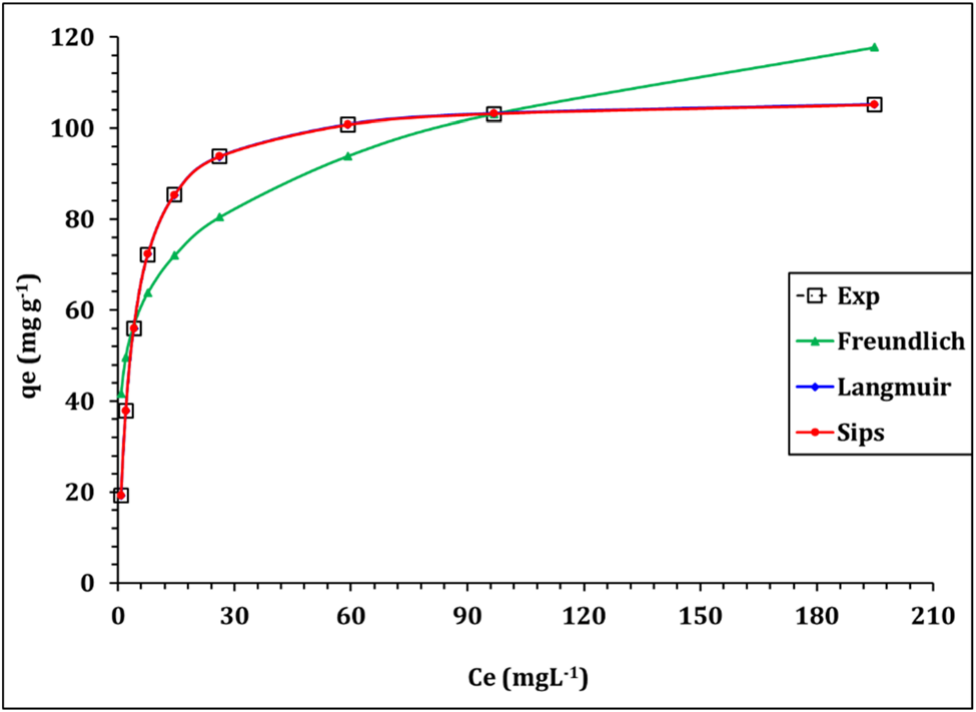
Isotherm profile for the adsorption of uranium(vi) ions from aqueous.

Equilibrium isotherm analysis is essential to describe how uranium ions distribute between the aqueous phase and the solid surface of the Zr-GA at equilibrium. It provides fundamental insight into adsorption capacity, surface heterogeneity, and the nature of binding interactions between sorbate and adsorbent. In this study, the experimental equilibrium data were fitted to three widely used isotherm models: Langmuir, Freundlich, and Sips. The non-linear forms of these equations and fitting parameters are provided in Table S1, while the model fits are illustrated in Fig. 10 and the corresponding constants summarized in Table 4.

**Table 4:** Parameters of the applied isotherm models

Langmuir model	
*q*_m_ (mg g^−1^)	107.2
*k*_L_ (L mg^−1^)	0.267
*R* ^2^	0.99
**Freundlich model**	

1/*n*_F_	0.2
*k*_F_ (mg g^−1^) (mg L^−1^)	43.3
*R* ^2^	0.84
**Sips model**	

*q*_S_ (mg g^−1^)	107.1
*k*_S_ (L mg^−1^)	0.268
*m*_S_	1.00
*R* ^2^	0.99

The Langmuir isotherm model assumes monolayer adsorption onto a homogeneous surface with energetically equivalent binding sites and no interaction between adsorbed molecules. A high correlation coefficient (*R*^2^ = 0.99) and low error values suggest that this model describes the system accurately. The calculated maximum adsorption capacity (*q*_max_) was 107.2 mg g^−1^, and the Langmuir constant (*k*_L_), representing the binding strength, was 0.267 L mg^−1^. Taken together, these parameters—particularly the relatively high q_max_—indicate strong uptake ability and are consistent with a high density of accessible, energetically similar sites on the Zr-GA surface; the *q*_max_ value compares favorably with ranges reported for U(vi) adsorption on a variety of engineered adsorbents, including carbonaceous and inorganic hybrid materials.^45,46^ The monolayer adsorption behavior supports the chemisorption-dominated mechanism identified in the kinetic modeling (Section 3.3.1) and is further substantiated by FTIR and XPS analysis indicating specific interactions between uranyl ions and carboxylate/hydroxyl groups (Sections 3.1.2 and 3.1.6).^32–34^ An important dimensionless parameter derived from the Langmuir equation is the separation factor (*R*_l_), which indicates adsorption favorability. The *R*_l_ values, calculated for concentrations ranging from 20 to 300 mg L^−1^, fall between 0.012 and 0.157 (Figure S3), confirming that U(vi) adsorption onto Zr-GA is highly favorable across a broad concentration range (since 0 < *R*_l_ < 1).^45,46^

In contrast, the Freundlich model, which assumes heterogeneous surface adsorption and multilayer interactions, produced a poorer correlation (*R*^2^ = 0.84). The calculated Freundlich constants were *K*_F_ = 43.3 mg g^−1^·(mg L^−1^)^−1^/*n* and 1/*n* = 0.20, which suggest favorable adsorption but highlight a deviation from surface heterogeneity assumptions. The low 1/*n* value reflects strong binding affinity, but the weaker model fit reinforces the notion that the Zr-GA surface is more homogeneous than heterogeneous.^32–34^ The Sips isotherm model, which merges features of both Langmuir and Freundlich equations, is suitable for systems that exhibit mixed site energies. The Sips model also demonstrated an excellent fit (*R*^2^ = 0.99), with parameters *q*_s_ = 107.1 mg g^−1^, *k*_s_ = 0.26 L mg^−1^, and a heterogeneity index *m*_s_ = 1.0. The value of *m*_s_ = 1.0 effectively reduces the model to Langmuir form, further confirming the monolayer, uniform energy site distribution on the sorbent surface.^32–34^

Together, these findings confirm that uranium adsorption onto Zr-GA occurs through strong, site-specific interactions across a well-defined monolayer. The high adsorption capacity and excellent fit to both Langmuir and Sips models underscore the precision of the sorbent's surface uniformity—attributes that stem from the rational coordination of zirconium clusters with gallic acid linkers during hydrothermal synthesis.

In conclusion, the isotherm modeling confirms that U(vi) adsorption onto Zr-GA is best described by the Langmuir and Sips models, indicative of monolayer chemisorption on a homogeneous surface. The sorbents's high capacity, strong affinity, and excellent model conformity highlight its practical potential in uranium remediation. These findings are in strong agreement with the structural and functional characteristics of the material, particularly its uniform porosity and abundance of oxygen-donor functional groups, which facilitate site-specific adsorption.

A comparative evaluation of U(vi) adsorbents (Table 5) clearly demonstrates the superior performance of the synthesized Zr-GA. With a maximum U(vi) uptake capacity of 107.2 mg g^−1^ achieved under mild conditions (pH 3.0, 25 °C, 120 min), the Zr-GA outperforms a wide range of reported materials, including biomass-derived, carbonaceous, inorganic, and polymeric adsorbents. For example, bio-based adsorbents such as activated moringa seed waste (ACMOSW@Ox) and modified rice stem exhibit capacities of 29.85 mg g^−1^ and 18.00 mg g^−1^, respectively, while requiring longer contact times (90–180 min) and operating at similar pH values.^51,52^ Engineered polymer-based sorbents such as CPAAm-EDA-CS_2_ and its derivative CPAm-DETA-CS_2_ show moderate capacities (∼57.8–60.2 mg g^−1^) under standard conditions.^2^ Similarly, PVC–NHA achieves 63 mg g^−1^ in just 15 min but at a higher operational pH (3.5).^53^ Despite reasonable performance, these materials generally suffer from lower binding affinities or narrower concentration ranges.

**Table 5:** Comparison of adsorption performance of U(vi) for different carbon adsorbents

Sorbent type	Time, min	Temperature, °C	pH	Initial concentration, mg L^−1^	Adsorption capacity, mg g^−1^	ref
Activating moringa seed waste (ACMOSW@Ox)	90	25	3	50–600	29.85	51
Silica modified with rhodamine-B	120	25	5	20–1000	35.00	54
Polyvinyl chloride –thiosemicarbazide(PVC-TSC)	30	25	4.5	20–150	30	55
Pyrolysis to produce bio-char species (PBC) PBC-Zn	120	25	4.5	20–80	21.9	56
CPAAm-EDA-CS2	60	25	4	20–120	57.8	2
CPAm-DETA-CS2					60.2	
Polyvinyl chloride-based *N*-hydroxyl amine (PVC-NHA)	15	25	3.5	20–150	63	53
Modified rice stem	180	25	4	5–60	18	52
SFeS@Biochar	300	25	4.19	20–100	76.32	57
Zr-GA adsorbent	120	25	3	20–300	107.2	Present work

Adsorbents such as SFeS@Biochar and PBC-Zn biochar demonstrate improved capacity (up to 76.32 mg g^−1^) but require significantly longer contact times (300 min) and are less efficient in strongly acidic environments.^56,57^ Functionalized silica materials, such as rhodamine-B-modified silica, offer moderate capacity (35 mg g^−1^) across a wide concentration range but again operate best at higher pH (pH 5).^54^ Among all these, the Zr-GA combines high capacity, rapid kinetics, and tolerance to acidic conditions, setting it apart as a next-generation uranium adsorbent. This exceptional performance is attributed to the synergistic combination of the high-affinity zirconium centers and oxygen-rich gallic acid ligands, which offer abundant and uniformly distributed adsorption sites. Furthermore, the material exhibits monolayer, homogeneous adsorption behavior (as confirmed by Langmuir and Sips models), supporting its selective and high-efficiency interaction with uranyl ions even in low-pH nitrate-rich systems. The hydrothermal synthesis approach is also straightforward, reproducible, and scalable, enhancing its practical appeal for industrial uranium recovery and wastewater remediation applications.

### 3.5. Thermodynamic investigation

**Fig. 11:**
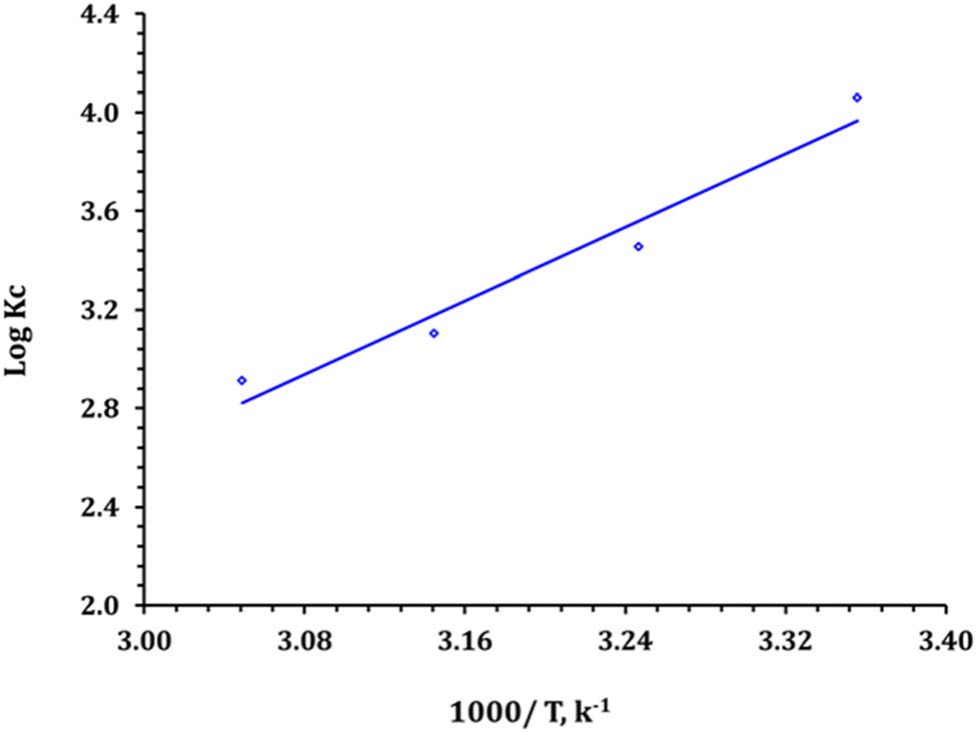
Thermodynamic profile for the capture process.

Thermodynamic analysis provides fundamental insights into the feasibility, energetic characteristics, and ordering behavior of U(vi) adsorption onto the Zr-GA. In this study, the standard Gibbs free energy change (Δ*G*°), enthalpy change (Δ*H*°), and entropy change (Δ*S*°) were evaluated based on temperature-dependent equilibrium studies performed at 298, 308, 318, and 328 K. These parameters were derived using the classical van't Hoff method, where the logarithm of the equilibrium constant (log *K*_c_) was plotted against the reciprocal of temperature (1/T).^35,58^ The relevant equations are provided in Table S1, and the van't Hoff plot is shown in Fig. 11.

As summarized in Table 6, all Δ*G*° values were negative, ranging from −23.2 to −18.3 kJ mol^−1^, confirming that the adsorption process is thermodynamically spontaneous across the entire temperature range investigated.^35,58^ The decreasing magnitude of Δ*G*° with rising temperature indicates that the spontaneity of the reaction declines slightly at elevated temperatures. This observation aligns well with the temperature-dependent adsorption results in Section 3.2, where uptake efficiency diminished with increasing thermal energy. The calculated enthalpy change (Δ*H*°) was −71.4 kJ mol^−1^, indicative of a strongly exothermic process. This value far exceeds the typical threshold for physical adsorption (<40 kJ mol^−1^), reinforcing that U(vi) uptake is driven by chemisorption, likely involving inner-sphere complexation with deprotonated carboxylate and hydroxyl groups present on the sorbent surface.^35,58^ Furthermore, the entropy change (Δ*S*°) was determined to be −162.0 J mol^−1^ K^−1^, reflecting a net loss in system randomness upon adsorption. This reduction in entropy is characteristic of systems where solvated ions become immobilized on solid matrices *via* specific coordination, resulting in more ordered interfacial structures.^47–49^

**Table 6:** The values of the thermodynamic parameters

Temperature, K	ΔG^o^ (kJ mol^−1^)	ΔH^o^ (kJ mol^−1^)	ΔS^°^ (kJ mol^−1^)
298	−23.2	−71.4	−162.0
308	−20.4		
318	−18.9		
328	−18.3		

These thermodynamic findings are in strong agreement with kinetic and isotherm modeling results. The spontaneous and enthalpically driven nature of the process, coupled with reduced interfacial disorder, supports a chemisorptive surface complexation mechanism, as also evidenced by FTIR and XPS data (Sections 3.1.2 and 3.1.6). Together, these results confirm that the uranium removal mechanism involves strong, site-specific interactions between uranyl ions and the oxygen-donor functionalities of the Zr-GA.

To sum up, the thermodynamic analysis clearly demonstrates that U(vi) adsorption onto Zr-GA is spontaneous, exothermic, and accompanied by a decrease in entropy, all of which are consistent with an ordered, ligand-controlled chemisorption process. These conclusions not only corroborate the proposed adsorption mechanism but also emphasize the thermal and operational stability of the sorbent under realistic environmental and process-relevant conditions.

### 3.6. Proposed adsorption mechanisms

Understanding the interaction mechanism between uranium species and the Zr-GA is essential for explaining its high adsorption performance and guiding its application in real-world systems.^59,60^ The collective findings from structural characterization, kinetic modeling, isotherm analysis, and thermodynamic evaluation suggest that U(vi) uptake by Zr-GA follows a chemisorption-driven, multi-stage mechanism involving both surface-level interactions and internal diffusion.

In the early stages of adsorption, under optimized acidic conditions (pH 3.0), uranium exists primarily in the solution as UO_2_^2+^ and UO_2_(NO_3_)^+^ cations (Figure S1). These species are strongly attracted to the negatively charged surface of Zr-GA, which exhibits a zeta potential of −46.69 mV, facilitating rapid adsorption through electrostatic interactions. Spectroscopic analyses (FTIR and XPS) confirm the presence of oxygen-rich functional groups—including carboxyl and hydroxyl moieties—that act as active binding sites. These groups participate in ligand exchange and coordinate with uranyl ions, forming stable inner-sphere surface complexes.^60,61^ Specifically, the observed shifts in *ν*_as/*ν*_s(COO^−^) and the emergence/intensification of Zr–O–C features in FTIR, together with the post-adsorption changes in O 1s and U 4f environments in XPS, are consistent with partial dehydration of UO_2_^2+^ and direct coordination to O-donor sites (–COO^−^/^−^O–) on the linker and Zr–oxo nodes. The excellent agreement with the pseudo-second-order kinetic model further confirms that chemisorption governs the adsorption mechanism, involving strong valence interactions between uranium and surface ligands.^62,63^ As surface sites become increasingly occupied, the process transitions into a slower phase dominated by intraparticle diffusion. This is supported by the two-stage behavior observed in the Weber–Morris and Boyd diffusion models, which suggest that both film diffusion and internal pore transport contribute to the overall rate limitation.^62,63^ The sorbent's mesoporous architecture—with an average pore size of 3.17 nm and a BET surface area of 37.94 m^2^ g^−1^—facilitates the migration of uranyl ions deeper into the structure, where additional binding can occur on interior functional groups.^64^ Notably, no evidence of simple outer-sphere/ion-exchange–dominated uptake was observed; the spectral and kinetic signatures favor inner-sphere coordination as the primary pathway.

Equilibrium data reinforce this mechanistic interpretation. The superior fit to the Langmuir and Sips isotherm models (*R*^2^ = 0.99) indicates monolayer adsorption onto energetically uniform active sites, consistent with chemisorptive binding. A high maximum capacity (107.2 mg g^−1^) and strong Langmuir affinity constant (*k*_L_ = 0.267 L mg^−1^) further support the specificity and strength of uranium–sorbent interactions.^45,46^ Thermodynamic parameters corroborate these findings: the process is spontaneous (Δ*G*° = −23.2 to −18.3 kJ mol^−1^) and strongly exothermic (Δ*H*° = −71.4 kJ mol^−1^), indicating that uranium uptake is energetically favorable and driven by enthalpy, not entropy. The negative entropy change (Δ*S*° = −162.0 J mol^−1^ K^−1^) suggests increased interfacial ordering as uranyl ions form structured complexes with the Zr-GA surface.^46^ A visual representation of the proposed adsorption mechanism is presented in Fig. S4, which outlines the sequence of events from electrostatic pre-concentration to inner-sphere complexation and intraparticle diffusion. The schematic highlights the coordinated role of the sorbents's negative surface charge, mesoporous architecture, and functionalized ligand environment in facilitating high-efficiency uranium removal from solution. From an HSAB perspective, the hard-acid character of U(vi) aligns with preferential coordination to hard O-donor sites (carboxylate/phenolate), rationalizing both the strong binding constants and the exothermic enthalpy of adsorption.

Altogether, these results point to a robust and synergistic adsorption mechanism. The process begins with rapid electrostatic attraction of uranyl cations to the highly negative surface, followed by inner-sphere complexation with oxygen donor groups. As adsorption progresses, intraparticle diffusion becomes increasingly important, enabling uranium capture within internal pore domains. This mechanism is consistent with the behavior of other high-performance uranium adsorbents, such as amidoxime-functionalized polymers, polyamidoamine-modified chelators, and Zr-based organic compounds like UiO-66 derivatives, all of which rely on multidentate oxygen- or nitrogen-based coordination for uranyl binding.^65,66^ The convergence of spectroscopic, kinetic, isotherm, and thermodynamic evidence demonstrates that Zr-GA offers a structurally uniform, chemically reactive, and diffusion-accessible platform for selective uranium adsorption. Its combination of fast initial uptake, strong surface complexation, and mesoporous diffusion pathways underscores its potential for real-world deployment in uranium recovery and wastewater treatment.

### 3.7. Desorption, reusability, and practical application

**Fig. 12:**
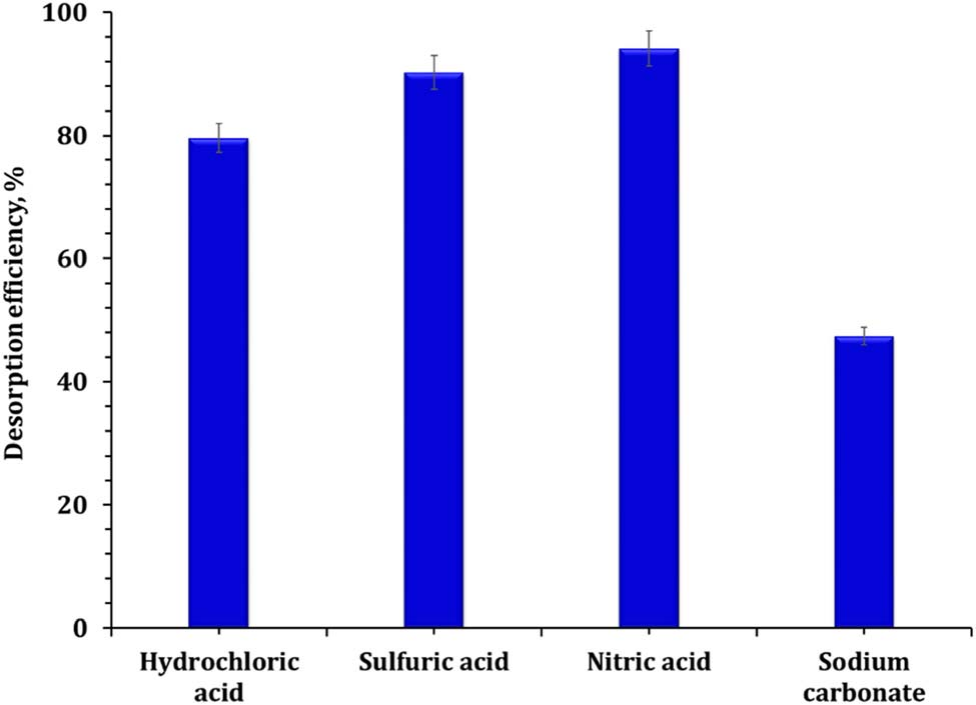
The desorption efficiency from the loaded adsorbent.

The practical application of adsorbents for environmental remediation depends not only on their initial performance but also on their chemical resilience and reusability over multiple adsorption–desorption cycles. To evaluate the regeneration potential of Zr-GA, desorption studies were conducted using various eluents under mild conditions. As illustrated in Fig. 12, 1.0 M nitric acid achieved the highest desorption efficiency at 94.1%, followed by sulfuric acid (90.2%) and hydrochloric acid (79.6%). In contrast, sodium carbonate was significantly less effective, achieving only 47.4%. These results suggest that uranium is strongly bound to the sorbent surface *via* inner-sphere complexation and that this binding can be efficiently reversed using proton-rich acidic eluents, particularly nitric acid.^60,61^

To further investigate operational durability, the Zr-GA underwent five successive adsorption–desorption cycles using 1.0 M HNO_3_ as the desorbing agent. As shown in Figure S5, the adsorption efficiency decreased only slightly, from 94.3% to 90.8%, while desorption efficiency remained consistently above 90% throughout the cycles. This minimal performance loss affirms the structural robustness and chemical stability of the adsorbent, supporting its use in continuous or cyclic treatment systems.

To assess the practical performance of the synthesized Zr-GA under realistic conditions, the adsorbent was applied to a real raffinate solution provided by the Nuclear Materials Authority (NMA). This effluent represents a typical uranium-bearing liquid residue generated during hydrometallurgical processing. Chemical analysis revealed that the sample contained U(vi) as the target metal ion, together with Fe(iii) and Ca(ii) as the only other constituents present at appreciable concentrations. No other cations or anions were detected in significant amounts. The raffinate also contained a higher nitrate concentration (0.5 M) compared to the laboratory model system (0.1 M), and the solution pH was slightly acidic (pH ≈ 3.0), closely matching the optimized conditions used in laboratory adsorption experiments, thereby minimizing uranyl hydrolysis and ensuring consistent comparison.

Despite the competitive presence of Fe(iii) and Ca(ii) ions, known to interfere with uranium adsorption *via* strong interaction with oxygen donor sites, and increased nitrate concentrations that promote uranyl–nitrate complexation, the Zr-GA exhibited a substantial uranium removal efficiency of approximately 91%. This result compares favorably to the 98% observed in a synthetic single-component system under the same conditions. This modest decrease reflects solution-matrix effects rather than a change in the adsorption mechanism, demonstrating that the adsorbent retains excellent selectivity toward U(vi) even in chemically competitive environments. The preferential uptake can be attributed to the strong affinity of uranyl ions (a hard Lewis acid) for the abundant oxygen-containing donor sites (–COOH and –OH) on the gallic acid linker, forming stable inner-sphere surface complexes with the Zr–O clusters. The rigid and negatively charged surface of the Zr-GA further enhances this selectivity by promoting electrostatic attraction and strong coordination with uranyl species. Additionally, only a minimal pH fluctuation (ΔpH < 0.3) was observed before and after adsorption, indicating both the chemical stability of the adsorbent and the absence of dissolution or side reactions in the raffinate medium. The integrity of the material was maintained after contact with the real solution, confirming its robustness and structural stability under realistic acidic conditions. Overall, the sustained high removal efficiency in the presence of elevated nitrate and competing metal ions highlights the strong selectivity, chemical resistance, and practical applicability of the Zr-GA for uranium removal from authentic industrial effluents.

## 4. Conclusion

This study demonstrates the successful design, synthesis, and application of a zirconium-gallic acid-based metal–organic compound (Zr-GA) as a selective and robust adsorbent for uranium(vi) removal from aqueous media. The sorbent structure was confirmed to possess mesoporosity and a chemically reactive surface populated with oxygen-bearing groups, particularly –COOH and –OH functionalities, which drive the adsorption process through surface complexation and diffusion mechanisms. Kinetic studies revealed a chemisorption-dominated mechanism, best described by the pseudo-second-order model, while isotherm modeling confirmed monolayer adsorption on energetically uniform sites with a maximum capacity of 107.2 mg g^−1^. The adsorption behavior was shown to be thermodynamically spontaneous and exothermic, accompanied by a decrease in system entropy—characteristics consistent with strong, specific uranyl– Zr-GA binding interactions. In addition to demonstrating excellent performance under controlled conditions, the Zr-GA maintained high reusability, achieving over 94% uranium recovery using dilute nitric acid and retaining more than 90% of its capacity after five cycles. Furthermore, the adsorbent exhibited high selectivity in a real radioactive liquid waste matrix containing competing metal ions, achieving 89.7% uranium removal without significant interference. These attributes—high capacity, selectivity, acid stability, and reusability—position Zr-GA as a promising candidate for uranium remediation in nuclear waste management, contaminated wastewater treatment, and selective recovery of strategic metals from complex aqueous environments.

## Conflicts of interest

The authors of this article would like to confirm that all of them have no conflict of interests with any organization or any person and the funding body is listed.

## Supplementary Material

RA-016-D5RA05880E-s001

## Data Availability

The datasets used and/or analyzed during the current study are available from the corresponding author on reasonable request. Supplementary information (SI): additional data and analyses supporting the main manuscript, including: U(vi) speciation *versus* pH (Hydra–MEDUSA) (Fig. S1); Arrhenius plot (Fig. S2); Langmuir separation factor (*R*_L_) (Fig. S3); schematic adsorption mechanism (Fig. S4); sorption/desorption cycles (Fig. S5); and supporting tables summarizing adsorption model equations (Table S1), surface charge/particle size before and after adsorption (Table S2), and Morris–Weber parameters at different temperatures (Table S3). See DOI: https://doi.org/10.1039/d5ra05880e.
