# Supplementary material for: Zr-gallic acid based metal organic compound as adsorbent for extraction of uranium(vi) from nitrate solution: adsorption behaviors and mechanisms
Source: RSC Adv. 2026 Jan 14;16(4):3057–76. doi: 10.1039/d5ra05880e (PMC12801241; doi:10.1039/d5ra05880e)
Supplement: RA-016-D5RA05880E-s001 [file RA-016-D5RA05880E-s001.pdf]

Supplementary Information (SI) for RSC Advances.
This journal is © The Royal Society of Chemistry 2026

Zr-Gallic acid based metal organic compound as adsorbent for extraction of Uranium (VI) from Nitrate solution: Adsorption behaviors and mechanisms

Ahmed M. Masoud

Nuclear Materials Authority, P.O. Box 530, El Maddi, Cairo, Egypt

Corresponding author: chemmaso010@hotmail.com

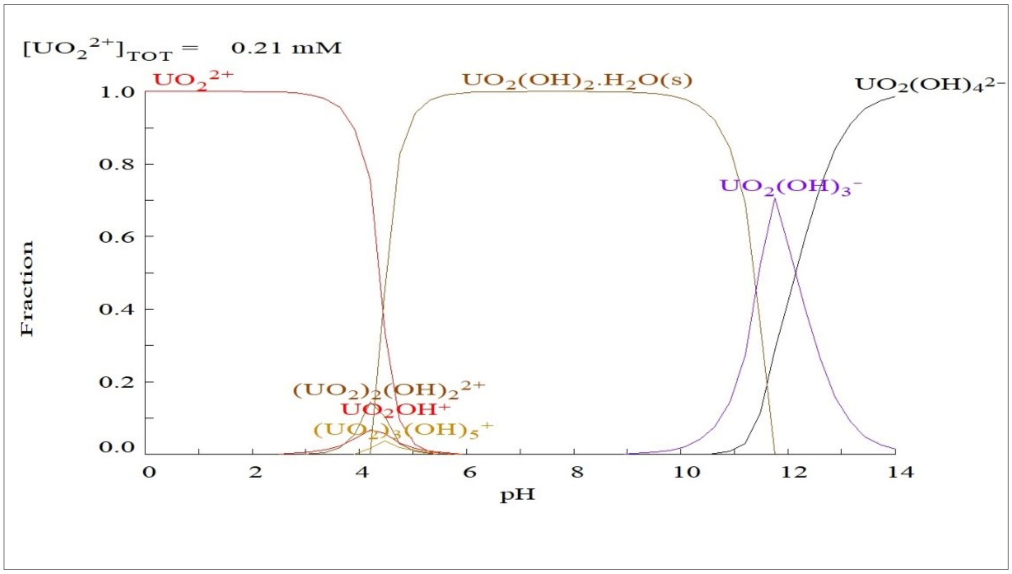

Figure S1: *U(VI) speciation as a function of solution pH, calculated using Hydra-MEDUSA equilibrium calculation program* ***[1]***.

1) *Hussein AEM, Taha MH (2013) Uranium removal from nitric acid raffinate solution by solvent immobilized PVC cement. J Radioanal Nucl Chem 295:709–715. https://doi.org/10.1007/S10967-012-2158-3/TABLES/2*

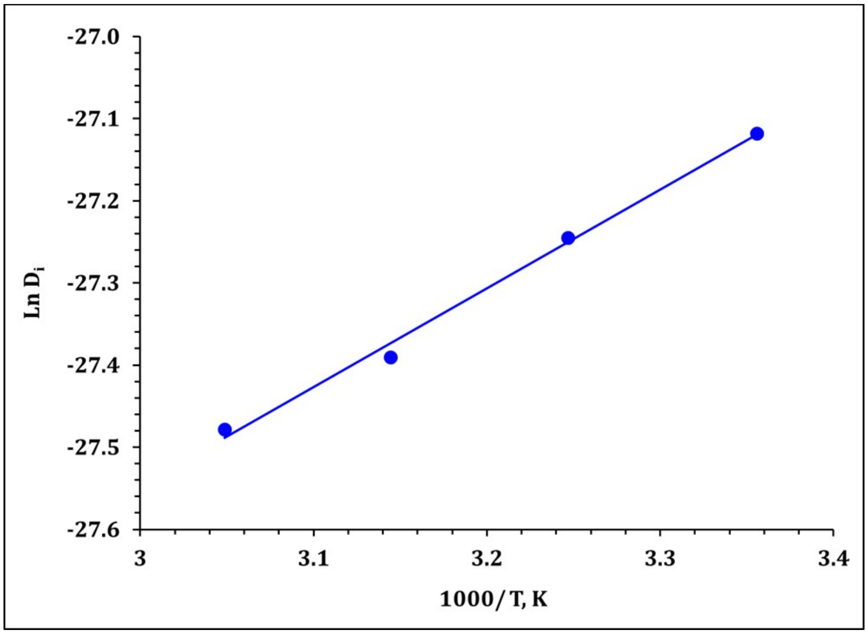

Figure S2: *Arrhenius plot for U(VI) sorption onto prepared composite*

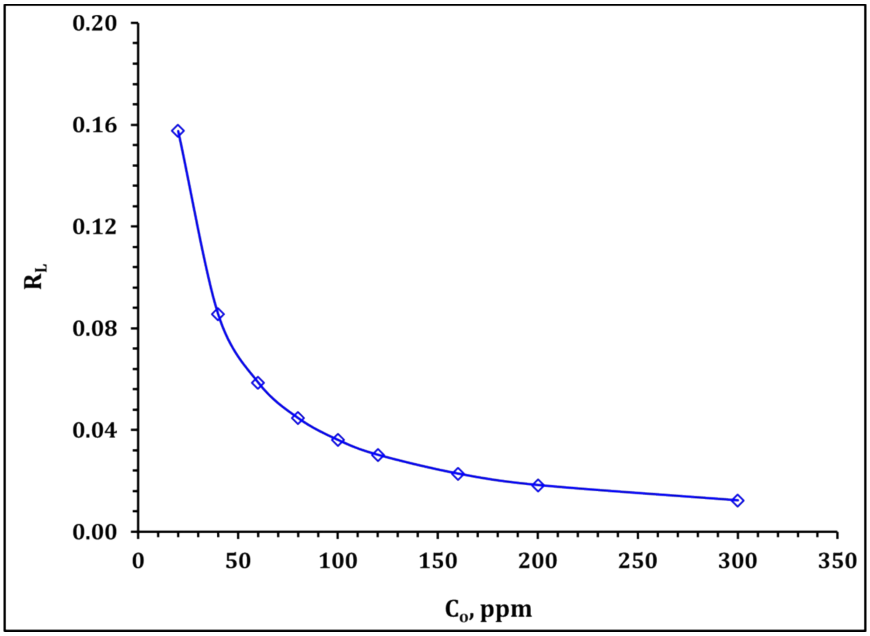

Figure S3: *Separation factor R_L of U(VI) adsorption process.*

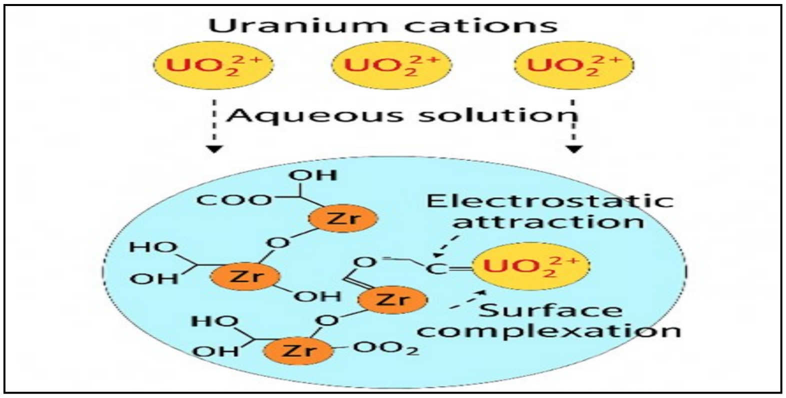

Figure S4. *Schematic representation of the proposed adsorption mechanism of U(VI) onto Zr-GA*

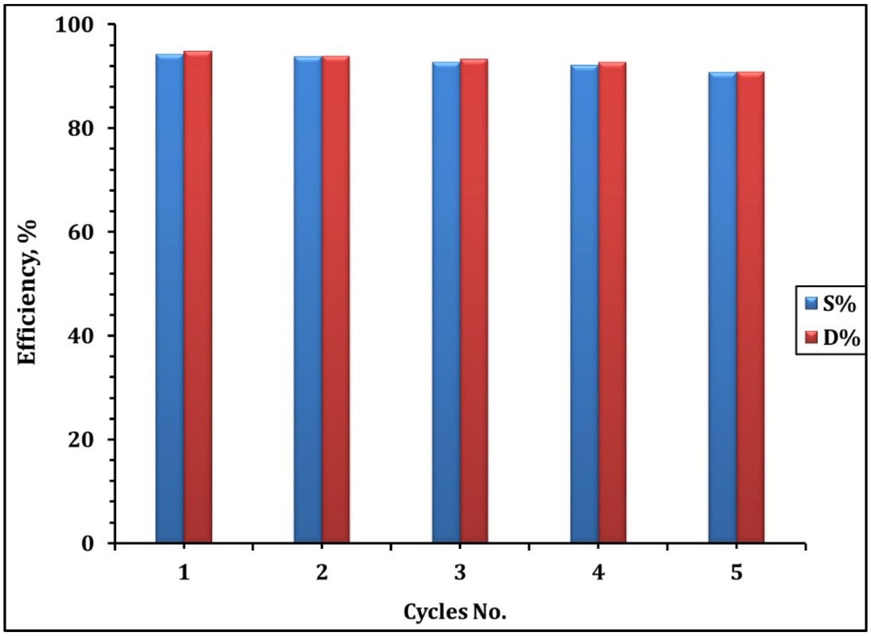

Figure S5: *Sorption/ desorption cycles for uranium capture process.*

Table S1: *Kinetic, isotherm, and thermodynamics equations for U(VI) adsorption process* ***[1-8]***.

Kinetics	Equations
**Pseudo-first-order**	$q_t = q_1\left(1 - e^{-k1t}\right)$
**Pseudo-second-order**	$q_t = \frac{1}{\left(1 \middle| k_2q_2^2\right) + \left(t \middle| q_2\right)}$
**Intra-particle diffusion model (IPD)**	$q_t = K_{id}t^{0.5} + C_i$
**Boyd model**	$Bt = -0.4977 - Ln(1 - F)$
**Isotherms**	**Equations**
**Langmuir model**	$q_e = \frac{q_m k_L C_e}{1 + k_L C_e}$
**Freundlich model**	$q_e = K_F C_e^{1/n_F}$
**Sips model**	$q_e = \frac{q_S\left(k_S C_e\right)^{mS}}{1 + \left(k_S C_e\right)^{mS}}$
**Thermodynamics**	**Equations**
	$\log K_C = -\frac{\Delta H^o}{2.303\,R} X \frac{1}{T} + C$ $-\Delta G^o = 2.303\,RT\log K_C$ $\Delta G^o = \Delta H^o - T\,\Delta S^o$
**Fitting**	**Equations**
**Coordination coefficient (R^2)**	$R^2 = 1 - \frac{\sum_1^n\left(q_{exp} - q_{pred}\right)^2}{\sum_1^n\left(q_{exp} - \bar{q}_{exp}\right)^2}$

qe (mg g^{-1}) is the equilibrium concentration of U(VI) species, and qt (mg g^{-1}) is the adsorbed amount of U(VI) species ions after time t (min), Ce (mg L^{-1}) is equilibrium concentration of U(VI) species. k_1 (min^{-1}) and k_2 (min^{-1}) are the rate constants for the pseudo first and second order, respectively. K_{id} (mg/g. $min^{0.5}$) is a rate constant, and C is the thickness of the boundary layer. F is

the ratio of the sorbed quantity at time t and that sorbed at equilibrium; B is the time constant and it equals $\pi^2 D_i / r_o^2$ **[4]**; Di is the effective diffusion coefficient of metal ions; ro is the radius of the solid particles; and n is an integer. q_m and q_S are the maximum sorption capacity (mg. g^{-1}) of Langmuir and Sips models. k_L (L. mg^{-1}), K_F (L/ mg), and K_S (L/ mg) are represent the constants of Langmuir, Freundlich, and Sips models. n refer to the sorption intensity, $\boldsymbol{q_s}$ is the theoretical isotherm saturation capacity (mg/g). K_C is a non-dimensional equilibrium constant and it equals K_d X 1000 X ρ **[5]**; T is the temperature (K), $\boldsymbol{R}$ is the universal gas constant (8.314 J mol $^{-1}$. K^{-1}), ρ is solution density g/ L, and C is a constant. R^2 is the coordination coefficient, the number of test points is n, the experimental equilibrium capacity is q_{exp} (mg g^{-1}), while the predicted capacity is q_{pred} (mg g^{-1}).

Table S2: *Surface charges and mean particle sizes of Zr-GA before and after adsorption process*

Analysis	***Before***	***After***
DLS analysis, nm	*549.5*	*1116.1*
Zeta potential, mV	*-46.69*	*-26.49*

Table S3: *The values of Morris-Weber model parameters.*

		**25 °C**	**35 °C**	**45 °C**	**55 °C**
**Weber and Morris model**	**k_i (mg/g min$^{1/2}$)**	1.06	0.88	0.67	0.44
	**C**	20.5	13.5	10.4	5.3
	**R^2**	0.98	0.97	0.98	0.96

References:

1) Hu, Q., Pang, S. and Wang, D., 2022. In-depth insights into mathematical characteristics, selection criteria and common mistakes of adsorption kinetic models: A critical review. *Separation & Purification Reviews*, *51*(3), pp.281-299.
2) González-López, M.E., Laureano-Anzaldo, C.M., Pérez-Fonseca, A.A., Arellano, M. and Robledo-Ortíz, J.R., 2022. A critical overview of adsorption models linearization: methodological and statistical inconsistencies. *Separation & Purification Reviews*, *51*(3), pp.358-372.
3) Tho, P.T., Van, H.T., Nguyen, L.H., Hoang, T.K., Tran, T.N.H., Nguyen, T.T., Nguyen, T.B.H., Le Sy, H., Tran, Q.B., Sadeghzadeh, S.M. and Asadpour, R., 2021. Enhanced simultaneous adsorption of As (iii), Cd (ii), Pb (ii) and Cr (vi) ions from aqueous solution using cassava root husk-derived biochar loaded with ZnO nanoparticles. *RSC advances*, *11*(31), pp.18881-18897.
4) Hassan, H.S., El-Kamash, A.M. and Ibrahim, H.A.S., 2019. Evaluation of hydroxyapatite/poly (acrylamide-acrylic acid) for sorptive removal of strontium ions from aqueous solution. *Environmental Science and Pollution Research*, *26*, pp.25641-25655.
5) Chen, X., Hossain, M.F., Duan, C., Lu, J., Tsang, Y.F., Islam, M.S. and Zhou, Y., 2022. Isotherm models for adsorption of heavy metals from water-a review. *Chemosphere*, *307*, p.135545.
6) Majd, M.M., Kordzadeh-Kermani, V., Ghalandari, V., Askari, A. and Sillanpää, M., 2022. Adsorption isotherm models: A comprehensive and systematic review (2010−2020). *Science of The Total Environment*, *812*, p.151334.
7) Taha, M.H., 2021. Sorption of U (VI), Mn (II), Cu (II), Zn (II), and Cd (II) from multi-component phosphoric acid solutions using MARATHON C resin. Environmental Science and Pollution Research, 28(10), pp.12475-12489.
8) Ebelegi, A.N., Ayawei, N. and Wankasi, D., 2020. Interpretation of adsorption thermodynamics and kinetics. *Open Journal of Physical Chemistry*, *10*(3), pp.166-182.
